# COVID-19 Antibody Seroconversion in Cancer Patients: Impact of Therapy Cessation—A Single-Center Study

**DOI:** 10.3390/vaccines11111659

**Published:** 2023-10-30

**Authors:** Lina Souan, Hikmat Abdel-Razeq, Sura Nashwan, Sara Al Badr, Kamal Alrabi, Maher A. Sughayer

**Affiliations:** 1 Department of Pathology & Laboratory Medicine, King Hussein Cancer Center, Amman 11941, Jordan; lsouan@khcc.jo (L.S.); sn.16562@khcc.jo (S.N.); sa.14588@khcc.jo (S.A.B.); 2 Department of Medicine, King Hussein Cancer Center, Amman 11941, Jordan; habdelrazeq@khcc.jo (H.A.-R.); ka.10798@khcc.jo (K.A.); 3 School of Medicine, The University of Jordan, Amman 11941, Jordan

**Keywords:** COVID-19, Pfizer BioNTech (BNT162b2), mRNA vaccine, BBIBP-CorV-virus, inactivated vaccine, IgG antibody titer, SARS-CoV-2, cancer patients, active cancer therapy

## Abstract

Background: The effective development of COVID-19 vaccination has mitigated its harm. Using two laboratory methods, we investigated the efficacy of the BNT162b2 mRNA and BBIBP-CorV COVID-19 vaccines on seroconversion rates in cancer patients undergoing active cancer treatment. Methods: SARS-CoV-2 vaccines were scheduled for 134 individuals. The consenting participants submitted three venous blood samples. Three samples: T0, T1, and T2. The ABBOTT-SARS-CoV-2 IgG II Quant and Elecsys^®^ Anti-SARS-CoV-2 assays were used to evaluate the samples and convert the antibody titers to WHO (BAU)/mL units. Results: Cancer patients exhibited a higher seroconversion rate at T2, regardless of vaccination type, and the mean antibody titers at T1 and T2 were higher than those at T0. BBIBP-CorV patients required a booster because BNT162b2 showed a higher seroconversion rate between T0 and T1. Statistics indicate that comparing Abbott and Roche quantitative antibody results without considering the sample collection time is inaccurate. Conclusions: COVID-19 vaccines can still induce a humoral immune response in patients undergoing cancer-targeted therapy. The strength of this study is the long-term monitoring of antibody levels after vaccination in cancer patients on active therapy using two different immunoassays. Further multicenter studies with a larger number of patients are required to validate these findings.

## 1. Introduction

Coronavirus disease-19 (COVID-19) is caused by the severe acute respiratory syndrome coronavirus 2 (SARS-CoV-2) [1]. SARS-CoV-2 infection leads to respiratory illnesses ranging from mild to severe symptoms, characterized by hypoxia, pneumonia [2], respiratory failure, immune dysregulation, cytokine storm, thromboembolic events [3], multi-organ failure, and death [4,5]. Smoking, hypertension, and other comorbidities were reported as risk factors for severe COVID-19 disease [6,7,8]. The World Health Organization (WHO) reported 758 million cases worldwide [9] and 1.74 million in Jordan [10]. Thus, the urgent development and distribution of vaccines have become the highest priority. The WHO gave the green light on the emergency use of the COVID-19 vaccine Pfizer/BioNTech in December 2020 [11], making it the first COVID-19 vaccine to get such approval. The “BBIBP-CorV” vaccine subsequently received approval for emergency use on 7 May 2021 [12]. Vaccines manufactured by Pfizer/BioNTech (Pfizer BioNTech (BNT162b2)) and BBIBP-CorV were first distributed in Jordan on 13 January 2021, to reduce the number of sick people requiring hospitalization and the severity of the adverse effects of COVID-19 [13].

The SARS-CoV-2 virus is a respiratory virus and a member of the genus Betacoronavirus in the Coronaviridae family. This family consists of positive-sense single-stranded RNA viruses enveloped by a capsid surrounded by a membrane [14]. The spikes that protrude from the envelope of the virus contain the receptor-binding domain (RBD) that binds to the angiotensin-converting enzyme2 (ACE2) receptor on the host cell membrane, which helps the virus to bind to host cells and mediate its entrance [15], making the spikes the keystone for viral infection. Therefore, they are the main targets of vaccines and the antibodies they produce [16]. 

Antibodies are proteins produced by the B-lymphocytes to fight the virus by neutralizing the spikes and preventing access to the host cells. The Pfizer BioNTech (BNT162b2) vaccine uses an mRNA that codes for the spike proteins, helping the host cell to create proteins similar to the SARS-CoV-2 spike proteins that activate immune cells and antibodies against these proteins, resulting in a competent immunity against the actual spikes of the virus [17]. The “BBIBP-CorV” vaccine is an inactivated virus that introduces the deadly virus to the body, allowing the immune system to recognize and activate the immune system without infecting the host cells [18]. 

Cancer patients, specifically those on active cancer treatments, lack a competent immune system, making them vulnerable in the face of such a disease. Previous studies have shown that cancer patients might be at a high risk for COVID-19 infection [19,20]. Furthermore, cancer patients infected with SARS-CoV-2 have a poor prognosis for the disease [21], making them ideal candidates for COVID-19 vaccination.

This study investigates the efficacy of BNT162b2 and BBIBP-CorV COVID-19 vaccines in terms of antibody production and seroconversion using two laboratory detection methods in cancer patients undergoing active cancer treatment at King Hussein Cancer Center in Jordan. 

## 2. Materials and Methods

### 2.1. Study Design

This prospective cohort study, conducted between November 2021 and July 2022, aimed to evaluate the efficacy of SARS-CoV-2 vaccines in adult cancer patients and assess the serological response as measured by antibody titers. The study also compared the performance of different assays used to detect antibodies. The KHCC-Institutional Review Board (IRB) approved the study, and all participants provided informed consent (21 KHCC 16 F). This study primarily focused on comparing the serological responses to the Pfizer BioNTech (BNT162b2) COVID-19 mRNA vaccine and Sinopharm (Beijing, China): Covilo (BBIBP-CorV) vaccines among adult cancer patients. Additionally, the performance of various serological assays for measuring antibody titers was assessed.

#### 2.1.1. Study Participants

We included 134 adult cancer patients scheduled to receive two doses of the BNT162b2 or the BBIBP-CorV vaccine. 

#### 2.1.2. Vaccine Administration

Most participants received at least two doses of the BNT162b2 or the BBIBP-CorV vaccine. After each dose, the participants were followed up to record vaccine side effects. These side effects were categorized into four groups:

1. No symptoms
2. Mild symptoms (e.g., mild fever, chills, or headache)
3. Moderate symptoms (e.g., fevers above 38 °C and shivers that require medication)
4. Severe symptoms leading to hospitalization.

#### 2.1.3. Serological Assessment

**Figure 1:**
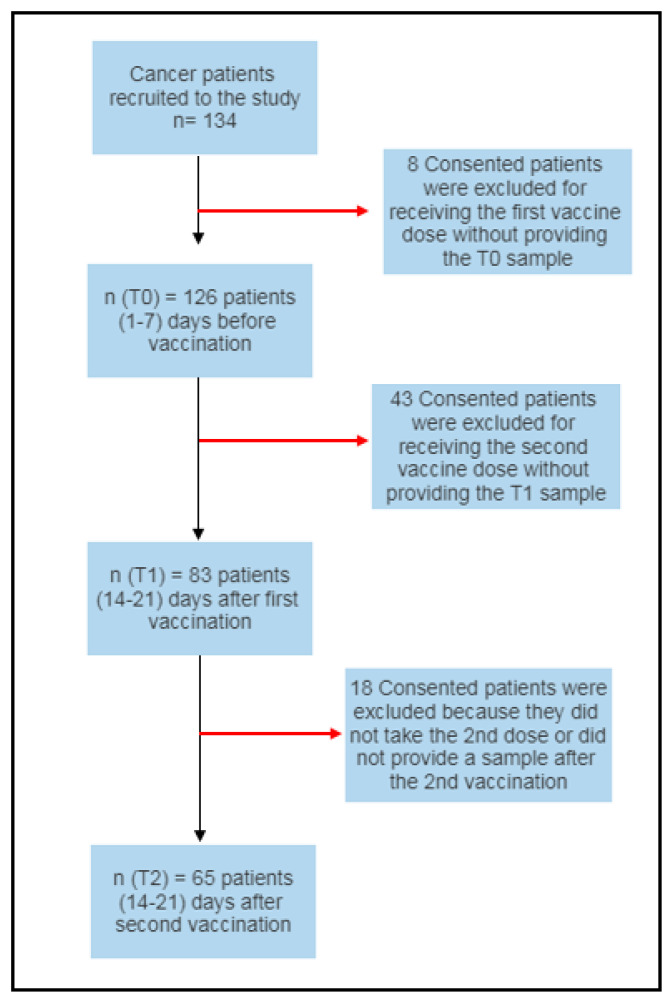
Schematic overview of the study design.

Blood samples were collected from all participants regularly to assess the vaccine’s serological response. Antibody titers were measured using two serological assays, and the results were compared. Three venous blood samples were obtained from each participant after obtaining consent. One 5 mL sample was drawn in a plain tube before the first dose of vaccination (T0), another sample was taken 14–21 days after the first vaccination (T1), and a third sample was drawn 14–21 days post-administration of the second vaccine dose (T2) (Figure 1). 

### 2.2. Antibody Response Testing

The IgG antibody response in the serum of cancer patients was evaluated using the SARS-CoV-2 IgG II Quant assay. This assay is a chemiluminescent microparticle immunoassay (CMIA) designed to quantify IgG antibodies to SARS-CoV-2, to the receptor-binding domain (RBD) of the S1 subunit of the spike protein (Abbott Architect SARS-CoV-2 IgG). According to the manufacturer’s instructions, the test was performed on an Architect i1000 analyzer (Abbott Diagnostics, Abbott Park, Chicago, IL, USA). The test results ranged from 21 to 40,000 AU/mL, with a positive result of ≥50 AU/mL and 100% favorable agreement with plaque reduction neutralization tests (PRNT) [22]. The antibody values were converted into WHO-binding antibody units per mL (BAU)/mL. The following conversion factor was used to calculate the WHO BAU/mL: 1 BAU/mL, corresponding to 0.142 Abbott AU/mL [22]. Serum samples were centrifuged at 4300 relative centrifugal force (rcf) for 15 min and stored at −80 °C until testing. The samples were gradually thawed at 4 °C for 24 h prior to analysis using an Architect i1000 analyzer. 

The patient samples were divided into two groups. One sample was tested using the ABBOTT-SARS-CoV-2 IgG II Quant assay, and the second sample was tested using the Elecsys^®^ Anti-SARS-CoV-2 assay. The Elecsys^®^ Anti-SARS-CoV-2 assay is an immunoassay for the in vitro quantitative determination of total antibodies against the SARS-CoV-2 S protein RBD in human serum. Utilizing a recombinant RBD protein in a double-antigen sandwich assay format, this assay facilitated the quantitative determination of SARS-CoV-2 antibodies with high affinity. The test aims to evaluate the adaptive humoral immune response to the SARS-CoV-2 S protein after natural infection or in vaccine recipients. The samples were analyzed using a Cobas e801 (Roche Diagnostics GmbH, Mannheim, Germany). Elecsys^®^ Anti-SARS-CoV-2 S assay results ≥ 0.8 U/mL were considered positive [23]. 

The antibody titer values from the Roche analyzer were converted into WHO binding antibody units (BAU)/mL to compare the antibody titer values from the ABBOTT system with the Roche system. The following conversion factors were used to calculate WHO BAU/mL: 1.0 BAU/mL corresponds to 1.0 Roche U/mL [22].

### 2.3. Seroconversion and Breakthrough Cases

Patients were observed for two months, during which any incident cases of COVID-19 breakthrough disease were reported. The seroconversion status of the patients who tested negative for cancer was also examined.

### 2.4. Statistical Analysis

The categorical data were expressed as frequency and percentage, and scale data were defined as the mean and standard deviation. Moreover, a marginal homogeneity test was used to determine the symptom degree and proportion differences between the first and second doses of the BNT162b2 vaccine. Repeated measures ANOVA was utilized to investigate the antibody titer mean differences at three time points using the Roche and Abbott assays. Furthermore, Cochran’s Q test was used to assess cancer patients’ anti-COVID-19 seroconversion rate. Point biserial correlation was used to correlate the patients’ age and sex with the antibody titer. The mixed ANOVA model was used to compare the efficacy of the vaccine type in generating anti-COVID-19 antibody titers at the three measurement times. The interclass correlation was used to investigate the agreement level of antibody titers between the Roche and Abbott assays at the three measurement times. A *p*-value set at <0.05 was considered statistically significant, and SPSS-IBM software version 28 was used to analyze the data.

## 3. Results

### 3.1. Patients’ Demographics

This study included 134 cancer patients. However, 65 patients committed to giving blood samples at the designated time intervals. Of these patients, 54 (83.1%) were females and 11 (16.7%) were males. The mean age ± SD of the patients was 53.2 ± 12.6 years. There were 59 patients (90.8%) with solid cancer and six patients (9.2%) with hematological cancer. The BNT162b2 vaccine was administered to 46 (70.8%) cancer patients, while 19 (29.2%) patients took the BBIBP-CorV (Table 1).

Of these patients, 43 (66.15%) were on active cancer treatment before vaccination and when drawing the first blood sample. The remaining 22 patients (33.8%) had stopped their therapies at least three months before vaccination. The types of active treatment were divided into the following subgroups: eight (12.3%) patients were on hormonal therapy. One patient (1.5%) received immunotherapy, and the other (1.5%) received radiotherapy. Of these patients, 33 (50.8%) received active chemotherapy treatment (Table 2). 

### 3.2. The Severity of Vaccine-Induced Side Effects after the First and Second Vaccine Doses 

Statistical analysis of data from patients who gave blood samples at all three-time points (n = 65) revealed no significant relationship between symptom severity and vaccine type at T1 and T2 (*p* > 0.05), as evidenced by the chi-square test (Table 3).

### 3.3. Evaluation of the Anti-COVID-19 Seroconversion Rate in Cancer Patients

To investigate the antibody seroconversion rate between the three times, we used Cochran’s Q test on patients’ data who were committed to giving blood samples at the three time points (n = 65). The analysis showed statistically significant differences among the three time points (*p* < 0.001). Detailed analysis using the post hoc test showed that the mean antibody titer at T0 was significantly lower than that at T1 and T2 (44 positive patients at T0 compared with 54 patients at T1 and 62 patients at T2, *p* = 0.012 and *p* < 0.001, respectively). However, there was no significant difference between T1 and T2 seroconversion rates, *p* = 0.063.

When comparing the vaccine type effect on seroconversion rates, we found that BBIBP-CorV and BNT162b2′s vaccines showed statistically significant differences between T0 and T2 in the seroconverted cases. The number of positive seroconverted patients increased from 33 at T0 to 45 at T2 in BNT162b2 vaccinated patients (*p* = 0.008). Similarly, BBIBP-CorV vaccinated patients’ antibody titer increased from 11 at T0 to 17 positive patients at T2 (*p* < 0.001) (Table 4). 

On the other hand, no significant difference was observed between T1 and T2 in either of the vaccinated groups. For example, in the BNT162b2 vaccinated patients, the number of positive patients at T1 was 40, compared to 45 seroconverted-positive patients at T2 (*p* = 0.401). In the BBIBP-CorV-vaccinated group, the number of positive patients ranged from 14 at T1 to 17 at T2 (*p* = 0.231). Nevertheless, the BNT162b2 vaccine showed a significant difference in seroconversion rates between T0 and T1 (T0 = 33, T1 = 40 seroconverted patients, *p* = 0.040), unlike the BBIBP-CorV vaccinated group where the difference between T0 and T1 seroconverted patients was insignificant (T0 = 11, T1 = 14 seroconverted patients, *p* = 0.401). 

No statistical analysis was performed on the effect of cancer type on seroconversion rates due to insufficient sample size among hematologic cancer patients compared with solid tumor patients (6 vs. 59 patients, respectively).

### 3.4. Association of Seroconversion Rates with Cancer-Directed Therapy

We analyzed the data using the chi-square test to explore further the association between seroconversion rates to COVID-19 vaccines and the treatment type. Our data showed that there was no significant association between cancer-directed therapy and seroconversion rate after each vaccine dose, regardless of the type of vaccine (*p* > 0.05) (Table 5). 

However, three patients did not show any response to vaccination. Two had solid cancers, one had BNT162b2, the second had BBIBP-CorV vaccine, and both were on chemotherapy. While the third patient had a hematologic malignancy (follicular lymphoma), had received the BBIBP-CorV vaccine, and was not on active therapy.

### 3.5. Assess Cancer Patients’ Anti-COVID-19 Antibody Titer Responses with Gender

Studying the correlation of antibody titer to COVID-19 vaccines with sex using point biserial correlation statistical analysis, we found no significant association at T1. At the same time, males reported a significantly lower titer than females at T2 (r = −0.249, *p* = 0.045).

### 3.6. Assess Cancer Patients’ Anti-COVID-19 Antibody Titer Responses

**Figure 2:**
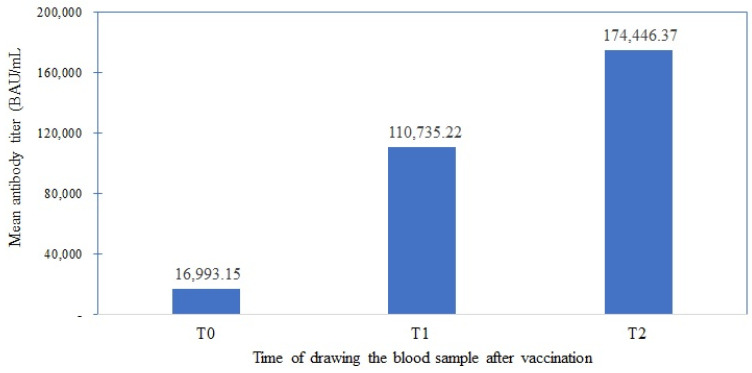
Mean antibody titer of SARS-CoV-2 in cancer patients (BAU/mL).

A repeated measures ANOVA was utilized to investigate antibody titer mean differences at the three time points using the Abbott SARS-CoV-2 IgG II Quant assay after converting the units to the WHO units. The results showed that the sphericity assumption was violated. Hence, the Huynh-Feldt correction epsilon value was reported. The within-subject effect revealed a significant difference in the antibody mean titer across the three time points measured by Abbott assay F (1.532, 99.96) = 34.675, *p* < 0.001 (Figure 2). The LSD post hoc test for multiple comparisons showed that the T2 antibody titer (174,446.37 BAU/mL) was significantly higher than (T1) (110,735.22 BAU/mL) and T0 (16,993.15 BAU/mL), *p* < 0.001.

### 3.7. SARS-CoV-2 Breakthrough Infection Rates in Cancer Patients after Each Vaccine Dose

To explore the efficacy of the COVID-19 vaccines in inducing protection against SARS-CoV-2 virus breakthrough infection after the first or second vaccine dose, we compared data after the first BNT162b2 and BBIBP-CorV vaccine doses and the second dose. The results showed no significant association between breakthrough COVID-19 infection and vaccine type at T1 and T2 (*p* > 0.05), as evidenced by the chi-square test (Table 6).

Moreover, when using Point biserial correlation statistical analysis, our data demonstrated that patients who had lower IgG antibody titer had breakthrough COVID-19 infection after the first vaccine dose (5 positive patients with breakthrough infection, and their mean antibody titer was 11,591.85 BAU/mL). However, this correlation was insignificant (r = −0.123, *p* = 0.325). Similarly, there was an insignificant negative correlation between antibody titer and COVID-19 breakthrough infection after the second vaccine dose (4 positive patients with breakthrough infection and their mean antibody titer 3539.07 BAU/mL) r = −0.169, *p* = 0.175.

### 3.8. Correlation Versus Agreement Levels of IgG Antibody Titer Measured by Roche and Abbott Systems over the Three Times Points

Our data showed a strong positive significant correlation between antibody titers using Roche and Abbott at time zero, one, and two, regardless of the vaccine type. When both measures were correlated based on the COVID-19 vaccine types, a strong positive significant correlation was observed for both vaccines at the three measurement points (Table 7).

However, when the interclass correlation coefficients (ICC) and their 95% CI were computed based on absolute agreement with the two-way mixed effect model of the anti-COVID-19 antibody titer measured by either Roche or ABBOTT using WHO converted units, Table 8 reveals that there was good agreement between the two systems at T0, and moderate agreement at T1 and T2 [24].

When the qualitative results from both systems were compared using Cohen’s kappa for inter-rater reliability (Roche vs. Abbott), there was a moderate agreement between T0 and T1. However, there was no agreement at T2 (both classified cases randomly) (Table 9).

## 4. Discussion

SARS-CoV-2 continues to cause severe disease and death, particularly in cancer patients with compromised immune systems, who are more vulnerable to infection [25]. As a result, they are a high-priority category for the COVID-19 vaccine [26,27]. So far, the available evidence regarding the safety and effectiveness of COVID-19 vaccines for vulnerable populations is limited [28]. Hence, the purpose of this study was to assess the efficacy of the BNT162b2 and BBIBP-CorV COVID-19 vaccines in terms of antibody production and seroconversion in cancer patients undergoing active cancer treatment at three different time points using two laboratory detection methods: the Abbott and Roche automated systems. This comparison will assist physicians in interpreting SARS-CoV-2 antibody levels due to vaccination, allowing them to design or modify vaccination strategies.

In our study, 83.1% of the patients were females, and their average age was 53.2 ± 12.6 years. Patients with solid cancers were more predominant than those with hematological cancers (90.8% vs. 9.2%). In addition, most patients (70.8%) received the BNT162b2 vaccine, whereas (29.2%) received the BBIBP-CorV vaccine. The most frequent side effects after the second vaccine dose were mild symptoms (50 and 52.6%) in the BNT162b2 and BBIBP-CorV vaccines, respectively. This observation is similar to other reported studies in healthy individuals, where the adverse effects of BNT162b2 were transient and generally mild in healthy individuals [29]. Therefore, the COVID-19 vaccine should be widely recommended for cancer patients to mitigate the disease’s deadly effects.

Analyzing the antibody seroconversion rate data between the three time points showed that the mean antibody titer at both T1 and T2 was significantly higher than that at T0, similar to the results of studies in the healthy population [30]. Furthermore, cancer patients showed a significant increase in seroconversion at T2 after both vaccines, similar to the healthy controls reported in recent studies [30]. However, our data showed no significant difference in seroconversion rates between T1 and T2 in either of the vaccinated groups, consistent with the findings of Wheeler et al. study in their study of healthy individuals [31]. When the number of seroconverted patients at T0 and T1 was evaluated, our data revealed that BNT162b2 vaccinated patients had a considerably higher seroconversion rate between T0 and T1, which is consistent with earlier studies in healthy adults [32,33,34]. BBIBP-CorV vaccinated group, on the other hand, demonstrated an insignificant distinction between T0 and T1 seroconversion rates, similar to a study conducted in a healthy population by Zhang et al. [35], which indicates that the BBIBP-CorV vaccine requires a second dose or a booster vaccine dose to enhance the humoral immune response in cancer patients, as in healthy individuals.

In terms of the relationship between cancer treatment type and seroconversion rates to COVID-19 vaccinations, our findings revealed that there was no significant relationship between cancer-specific therapy and seroconversion rate after each vaccine dosage, independent of vaccine type, which is similar to previous studies in solid cancer patients vaccinated with the BNT162b2 vaccine [36]. This result indicated that cancer therapy did not affect the seroconversion rates to COVID-19 vaccination. It is worth mentioning, however, that three individuals did not respond to immunization, regardless of the treatment method. Two patients were diagnosed with solid tumors, while the third was diagnosed with a hematologic malignancy. This conclusion is consistent with previous studies that have demonstrated that certain cancer patients could not establish significant antibody responses after immunization [37]. Furthermore, our data showed that male cancer patients produced a significantly lower IgG antibody titer than females, similar to healthy healthcare workers reported in the literature [38]. Consistent with findings among healthcare professionals, our data showed that the RBD-specific IgG antibody titer of the S1subunit of the spike protein of SARS-CoV-2 was significantly higher at T2 [39], regardless of the vaccine type.

Furthermore, regardless of the vaccination type, we found a negligible negative correlation between IgG antibody titers and breakthrough COVID-19 infection after the first and second vaccine doses. This insignificant result could be attributed to the substantial variation in the sample size. Nonetheless, our findings are consistent with earlier studies in cancer patients, which found that antibody titers were strongly related to protection against breakthrough infections and severe illnesses [40].

We chose to investigate the correlation between Roche’s and Abbott’s analytical methods for assessing anti-SARS-CoV-2 IgG antibodies due to insufficient information on whether the relationship between the two antibody tests is robust. Similar to previous studies [41,42], our data revealed a strong positive correlation between antibody titers using the Roche and Abbott systems at all time points and for both vaccines at the three measurement points, as reported for healthy healthcare workers [42]. Moreover, there was a good agreement between the anti-COVID-19 antibody titer measured by Roche or ABBOTT using WHO-converted units at T0 and moderate agreement at T1 and T2 using interclass correlation coefficients (ICC). Hence, the observed high correlation between the two techniques suggests that the S-RBD antibody is kinetically stable in both assays and that these assays will assist with diagnostics, gauging vaccination efficacy, and determining the adaptive immune response to COVID-19 vaccines in cancer patients.

However, caution should be taken when comparing the qualitative data from both systems at different time points after immunization. For example, our results revealed that the Abbott and Roche systems had a moderate agreement at T0 and T1 but not at T2. As previously reported, the variance between both methods in antibody level kinetics depends on the blood sampling time and should be carefully acknowledged [43,44].

It is difficult for clinicians to determine the optimal time to administer vaccines or boosters to patients undergoing cancer-specific therapies. To give their patients time to be vaccinated against COVID-19, some doctors delay treatment, whereas others wait until the disease has been fully treated. Currently, more information is required to support these decisions. In this study, we demonstrated for the first time that cancer patients can establish a significant humoral immune response to the BNT162b2 and BBIBP-CorV vaccines despite undergoing cancer-targeted therapy.

Furthermore, when employing the Abbott or Roche assays to measure antibody responses to these vaccines in cancer patients, we demonstrated that the comparison of quantitative anti-spike SARS-CoV-2 antibody testing is significantly contingent on the timing of blood collection, which varies with time after both the first and second doses of the vaccine. As a result, comparing different quantitative SARS-CoV-2 antibody results without standardization of sample collection time does not appear acceptable or accurate, even after converting the units to standard WHO-BAU units [43].

Our findings support Ivanov et al.’s conclusion that the anti-SARS-CoV-2 antibody levels assessed by the ABBOTT and Roche systems are not linear [45].

## 5. Conclusions

The primary strength of this study was the side-by-side assessment utilizing two different immunoassays in cancer patients undergoing active cancer therapy, as well as the long-term follow-up observation of antibody titers after vaccination with COVID-19 mRNA and inactivated virus vaccines. The small size of the study sample was a significant limitation, as well as a large proportion of females compared to males. Nevertheless, clinicians can still use these results to evaluate SARS-CoV-2 antibody levels better and to plan future vaccination strategies.

## Figures and Tables

**Table 1:** Cancer patients’ demographic characteristics.

Variables	Categories	n	%
Gender	Male	11	16.9
	Female	54	83.1
Cancer category	Solid cancer	59	90.8
	Hematologic cancer	6	9.2
Type of vaccine	BNT162B2	46	70.8
	BBIBP-CORV	19	29.2
Age (years)	Mean ± SD	53.2 ± 12.6	

**Table 2:** The frequency of active cancer therapy treatment for cancer patients at sample acquisition.

Type of Active Cancer Therapy	n	%
No therapy	22	33.8
Hormonal Therapy	8	12.3
Immunotherapy	1	1.5
Radiotherapy	1	1.5
Chemotherapy ± other modalities *	33	50.8

* Patients on combined radiotherapy plus chemotherapy or combined hormonal therapy plus chemotherapy were added to the chemotherapy group.

**Table 3:** Severity of vaccine-induced side effects after the first and second vaccine doses.

Time Point	Vaccine Type	No Symptoms	Mild	Moderate Symptoms	Severe	*p*-Value
T1	BNT162b2 (n = 46)	14 (30.4%)	24 (52.2%)	7 (15.2%)	1 (2.2%)	0.222
	BBIBP-CorV (n = 19)	9 (47.4%)	10 (52.6%)	0 (0%)	0 (0%)	
T2	BNT162b2 (n = 46)	18 (39.1%)	23 (50%)	5 (10.9%)	0 (0%)	0.209
	BBIBP-CorV (n = 19)	8 (42.1%)	10 (52.6%)	0 (0%)	1 (5.3%)	

**Table 4:** Evaluation of the anti-COVID-19 seroconversion rate in cancer patients at T0 and T2 for both vaccines.

Vaccine	Time	Seroconversion Status			*p*-Value
BNT162b2	T0		Negative (n = 13)	Positive (n = 33)	
	T2	Negative (n = 1)	1	0	<0.001
		Positive (n = 45)	12	33	
BBIBP-CorV	T0		Negative (n = 8)	Positive (n = 11)	
	T2	Negative (n = 2)	2	0	<0.031
		Positive (n = 17)	6	11	

**Table 5:** Association of seroconversion rates with Cancer-directed therapy.

Cancer-Directed Therapy	T1			T2		
	Negative	Positive	*p*-Value	Negative	Positive	*p*-Value
	n (%)	n (%)		n (%)	n (%)	
No therapy (n = 22)	5 (22.7)	17 (77.3)	0.340	1 (4.5)	21 (95.5)	0.785
Chemotherapy (n = 35)	6 (17.1)	29 (82.9)		2 (5.7)	33 (94.3)	
Hormonal Therapy (n = 8)	0.0 (0)	8 (100.0)		0.0 (0)	8 (100.0)	

**Table 6:** Percent breakthrough infection after the first and second vaccine doses.

Time Point	Vaccine Type	Breakthrough COVID-19 Infection		*p*-Value
		No	Yes	
T1	BNT162b2	41 (89.1%)	5 (10.9%)	0.310
	BBIBP-CorV	19 (100%)	0 (0%)	
T2	BNT162b2	43 (95.6%)	2 (4.4%)	0.341
	BBIBP-CorV	17 (89.5%)	2 (10.5%)	

**Table 7:** Correlation between Roche and Abbott systems in antibody titer divided by vaccine type at three-time intervals.

Roche BAU/mL	Abbott BAU/mL	Vaccine Type	Spearman Rho	*p*-Value
T0	T0	BNT162b2 BBIBP-CorV	0.914 0.896	<0.001 <0.001
T1	T1	BNT162b2 BBIBP-CorV	0.914 0.800	<0.001 <0.001
T2	T2	BNT162b2 BBIBP-CorV	0.726 0.873	<0.001 <0.001

**Table 8:** Agreement levels (Interclass coefficient analysis) between Roche and Abbott antibody titer levels converted to WHO units over the three times of measurements.

Measurement Times	ICC/Average Measure	95% Confidence Interval	
		Lower Bound	Upper Bound
T0	0.765	0.610	0.857
T1	0.600	0.168	0.790
T2	0.602	0.117	0.799

<0.5 poor, 0.5–0.75 moderate, 0.75–0.90 good and >0.90 excellent reliability [24].

**Table 9:** Agreement levels (Cohen kappa for interrater reliability) between Roche and Abbott qualitative results over the three times of measurements.

Time Points	Cohen Kappa	*p*-Value	Interpretation
T0	0.598	<0.001	Moderate agreement
T1	0.616	<0.001	Substantial agreement
T2	0.024	0.825	None to slight agreement

Values ≤ 0 indicate no agreement and 0.01–0.20 as none to slight, 0.21–0.40 as fair, 0.41– 0.60 as moderate, 0.61–0.80 as substantial, and 0.81–1.00 as almost perfect agreement.

## Data Availability

Data are available upon request from msughayer@KHCC.JO.
